# A Case Report of Cor Triatriatum Sinister (CTS) in an Asymptomatic Adult with Chronic Adhesive Pericarditis

**DOI:** 10.2174/0115734056383803250523070650

**Published:** 2025-06-03

**Authors:** Yuan-Teng Hsu, Chee-Siong Lee, Jui-Sheng Hsu, Che-Lun Hsu, Ding-Kwo Wu

**Affiliations:** 1 Department of Medical Imaging, Kaohsiung Medical University Hospital, Kaohsiung Medical University, Kaohsiung, Taiwan; 2 Department of Radiology, Kaohsiung Medical University, Kaohsiung, Taiwan; 3 Department of Internal Medicine, Division of Cardiology, Kaohsiung Medical University Hospital, Kaohsiung Medical University, Kaohsiung, Taiwan

**Keywords:** Cor triatriatum sinister, Congenital cardiac abnormality, Atrial fibrillation, Pericardial calcification, Chronic adhesive pericarditis, Case report

## Abstract

**Introduction:**

Cor Triatriatum Sinister (CTS) is a rare congenital anomaly, accounting for 0.1%- 0.4% of congenital heart diseases. While often diagnosed and treated in infancy, some cases remain asymptomatic until adulthood due to large fenestrations. This report presents a unique case of CTS in an adult coexisting with chronic adhesive pericarditis, which may have contributed to chronic atrial dilatation, a condition not previously documented.

**Case Presentation:**

A 60-year-old asymptomatic Taiwanese male underwent a routine medical examination. Coronary computed tomography angiography revealed a fenestrated septum dividing the left atrium, consistent with CTS. Virtual endoscopy confirmed two wide fenestrations. Notably, chronic adhesive pericarditis, evidenced by curvilinear calcifications, was diagnosed. This condition likely exacerbated the hemodynamic impact of CTS, contributing to left atrial dilation and atrial fibrillation. Atrial fibrillation was identified, and the patient was treated with an anticoagulant for stroke prevention.

**Conclusion:**

This is the first reported case of CTS coexisting with chronic adhesive pericarditis. Advanced imaging modalities, including cardiac computed tomography, angiography, and virtual endoscopy, are crucial for diagnosis and anatomical evaluation. Chronic adhesive pericarditis may amplify the effects of CTS, leading to complications, including atrial fibrillation. Anticoagulation is essential for stroke prevention in such cases.

## 1 INTRODUCTION

Cor Triatriatum Sinister (CTS) is an extremely rare congenital anomaly, accounting for about 0.1%-0.4% of congenital heart diseases [1, 2]. Although most cases are diagnosed in infancy or early childhood and require surgical treatment, some patients may remain asymptomatic until adulthood due to a sufficiently large fenestration [3]. Asymptomatic adults with CTS may develop symptoms later in life, as the orifice of the membrane can undergo fibrosis or calcification, leading to obstruction. We herein present the case of a 60-year-old male with incidentally diagnosed CTS coexisting with chronic adhesive pericarditis. To the best of our knowledge, this combination has not been previously reported in the literature. In this case, prominent curvilinear calcifications along the pericardium were noted, consistent with chronic adhesive pericarditis. Chronic adhesive pericarditis reduces the elasticity of the pericardium, which may lead to left atrial enlargement. We believe this condition exacerbated the hemodynamic impact of CTS and potentially contributed to the development of atrial fibrillation. Some case reports have emphasized the importance of stroke prevention in patients with CTS; therefore, this patient was treated with Edoxaban [3-6].

## 2 CASE PRESENTATION

**Fig. (1):**
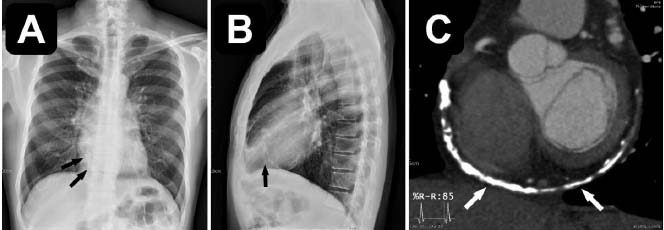
(**A**, **B**) Multiple curvilinear calcifications with variable thickness were noted along the contour of the right ventricle, extending into the right ventricular outflow tract and main pulmonary artery. (**C**) Thick curvilinear calcifications were noted in the posterior atrioventricular groove.

**Fig. (2):**
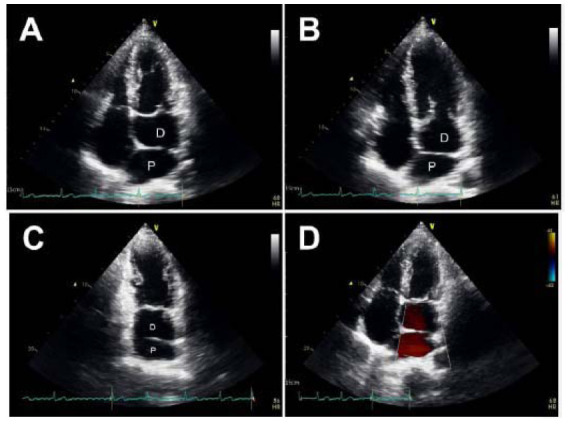
(**A**, **B**) Transthoracic cardiac sonography revealed an accessory septum at the left atrium, which divided the left atrium into a proximal chamber (P) and a distal chamber (D). (**C**) There was an obvious opening between the proximal chamber and the distal chamber. (**D**) Doppler echocardiography revealed no obvious turbulent flow in the left atrium.

**Fig. (3):**
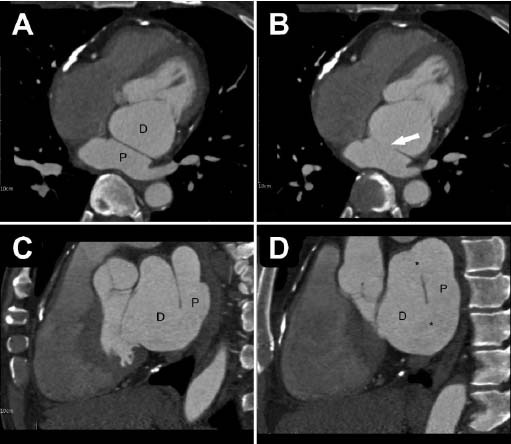
(**A**, **B**) The axial view of the cardiac computed tomography angiography revealed an accessory septum between the proximal chamber (P) and the distal chamber (D) in the left atrium. (**B**, **C**, **D**) Large openings in the accessory septum were noted in the axial and sagittal views. (white arrow and the asterisk).

**Fig. (4):**
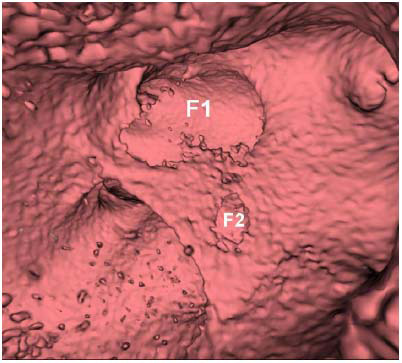
Virtual endoscopy images revealed two fenestrations on the accessory membrane in the left atrium (F1-2).

This was a 60-year-old asymptomatic Taiwanese male with no known medical history. He presented to our hospital for a comprehensive medical examination. He reported no family history of cardiovascular or genetic disorders and no relevant psychosocial or psychiatric conditions. Chest radiography showed a straightened left heart border, suggesting left atrial enlargement. No evidence of pulmonary vessel engorgement was observed. On chest radiography and computed tomography, multiple curvilinear calcifications of variable thickness were noted along the contour of the posterior atrioventricular groove and right ventricle, extending into the right ventricular outflow tract and main pulmonary artery (Fig. **1**). There was no known history of heart disease and no apparent symptoms of heart failure. These thick linear pericardial calcifications were consistent with chronic adhesive pericarditis. Transthoracic echocardiography revealed an incidental finding of an accessory septum in the left atrium with a large opening. There was no turbulent flow or regurgitation at the left atrium or mitral valve (Fig. **2**). The coronary computed tomography angiography demonstrated a fenestrated septum dividing the left atrium into two chambers, consistent with the diagnosis of Cor triatriatum sinister (Fig. **3**). Virtual endoscopy revealed two fenestrations connecting the proximal and distal chambers (Fig. **4**). No thrombi were detected in the left atrial appendage or left atrium. There was no associated atrial septal defect or ventricular septal defect. All four pulmonary veins drained into the proximal chamber. No evident adhesions, fibrosis, or calcification were observed at the anterior or posterior commissure of the mitral valve. The Electrocardiogram (EKG) showed atrial fibrillation with an average ventricular rate of 68 bpm (range 41~150 bpm), without ST segment or T wave changes. The Agatston score was zero, and the coronary artery anatomy was normal. To the best of our knowledge, this is the first case of a patient with CTS coexisting with chronic adhesive pericarditis. Although he remained asymptomatic, he had newly diagnosed atrial fibrillation and a CHA2DS2-VASc score of 1. For long-term prevention of stroke, he was prescribed Edoxaban 60 mg orally once daily for long-term stroke prevention.

## 3 DISCUSSION

Cor Triatriatum Sinister(CTS) is an extremely rare congenital anomaly, accounting for about 0.1%-0.4% of congenital heart diseases [1, 2]. It is characterized by the presence of a fibromuscular septum dividing the left atrium into proximal and distal chambers, referred to as the accessory and true left atrium, respectively [7]. The pulmonary veins typically drain into the accessory left atrium, while the true left atrium communicates with the left atrial appendage and mitral valve [8]. Variability in the septum’s size, shape, thickness, and location has been reported, and it can be complete, incomplete, or fenestrated in morphology [9]. Clinical symptoms depend on the size of the communication between the chambers and the presence of associated congenital anomalies [10].

CTS is usually diagnosed during infancy or early childhood, especially when the orifice is small. Symptoms may include pulmonary hypertension, poor growth, hemoptysis, orthopnea, or arrhythmias [7]. However, in cases with large fenestrations, diagnosis can be delayed until adulthood due to minimal hemodynamic disturbance [9, 11].

Transthoracic Echocardiography (TTE) is typically the first-line imaging modality, providing visualization of the intra-atrial membrane and assessment of blood flow. When further detail is required, Transesophageal Echocardiography (TEE) offers a better resolution of membrane morphology and fenestrations. Cardiac computed tomography angiography is a non-invasive technique that allows precise anatomical evaluation of the membrane, fenestrations, and associated cardiovascular anomalies [12, 13]. CTA also enables virtual endoscopy, offering a clear three-dimensional depiction of the intra-atrial membrane.

In the present case, CTA and virtual endoscopy identified a fenestrated membrane with two wide openings in the left atrium. CTS can be classified using systems such as the Loeffler, Lam, and Lucas classifications [14]. The Loeffler system, widely adopted due to its simplicity, categorizes CTS into three groups based on fenestration size: Group 1 (no openings), Group 2 (small openings), and Group 3 (wide openings) [3, 15]. Our patient was classified as Group 3, which may explain how he remained asymptomatic until the age of sixty, a rare clinical scenario [5, 11].

The etiology of CTS remains controversial [5, 6]. Several embryological theories have been proposed, including malincorporation of the common pulmonary vein into the left atrium [16, 17], abnormal growth of the septum primum (malseptation) [18], and external compression of the developing left atrium by a persistent left superior vena cava [19]. CTS frequently coexists with other congenital anomalies. In pediatric populations, Atrial Septal Defects (ASD) and anomalous pulmonary venous return are most commonly reported. Less frequent but significant associations include tetralogy of Fallot, double outlet right ventricle, and coarctation of the aorta [20]. Cor Triatriatum Dextrum (CTD), the right-sided counterpart of CTS, is even rarer. It results from the persistence of the right sinus venosus valve, leading to the partitioning of the right atrium, and is often associated with ASD or Ebstein’s anomaly [21]. CTS must be differentiated from a supramitral ring. In CTS, the left atrial appendage is part of the distal chamber connected to the mitral valve, whereas in the supramitral ring, the appendage lies within the proximal chamber, which receives the pulmonary veins [11]. CTS in adults is often associated with mitral regurgitation and atrial fibrillation. Asymptomatic individuals may develop symptoms later in life, possibly due to the progressive fibrosis and calcification of the septum. This process can lead to narrowing and obstruction of the septum fenestration, with symptoms similar to mitral stenosis. Additionally, age-related mitral regurgitation may contribute to atrial fibrillation [6, 18].

**Fig. (5):**
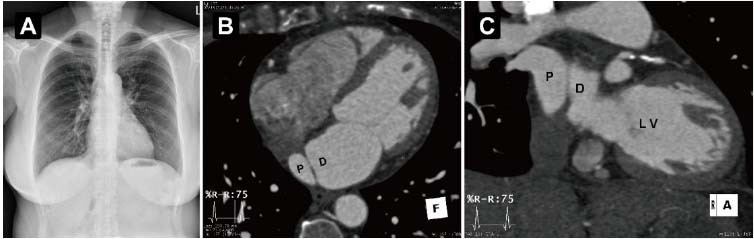
Comparison imaging from a patient with Cor Triatriatum Sinister (CTS) but without chronic adhesive pericarditis. (**A**) Chest radiograph showing no evident pericardial calcification. (**B**, **C**) Cardiac computed tomography angiography revealing an accessory septum dividing the left atrium into proximal (P) and distal (D) chambers. LV indicates the left ventricle.

In our patient, the septum did not demonstrate fibrosis or calcification. Instead, he had extensive pericardial calcification and pericardial thickening, consistent with chronic adhesive pericarditis, without any prior history or symptoms [22]. The diagnosis was incidental, as the patient exhibited no clinical symptoms indicative of structural heart disease, making the coexistence of CTS and chronic adhesive pericarditis an unexpected finding. To the best of our knowledge, no prior reports have described the coexistence of CTS and chronic adhesive pericarditis, underscoring the rarity of this presentation. For comparison, we additionally included imaging from another patient with CTS but without chronic adhesive pericarditis (Fig. **5**) to illustrate the imaging differences.

Chronic adhesive pericarditis may be clinically silent but can increase left atrial pressure by reducing pericardial compliance. This, in turn, may exacerbate left atrial dilation and contribute to atrial fibrillation [23]. In this case, we believe that the chronic adhesive pericarditis intensified the hemodynamic burden imposed by CTS, ultimately leading to atrial fibrillation.

Surgical resection of the intra-atrial fibromuscular membrane remains the definitive treatment for symptomatic CTS. When performed early, especially in patients without associated congenital defects, surgical outcomes are excellent [7, 9]. As our patient remained asymptomatic, surgical intervention was not indicated. Currently, there is no definitive evidence regarding how chronic adhesive pericarditis may impact surgical outcomes in CTS. However, we speculate that extensive pericardial calcification could increase surgical complexity. Similarly, should catheter ablation for atrial fibrillation be considered in the future, pericardial thickening may pose challenges to electrical conduction or procedural access, although this remains speculative.

Strokes have been reported in CTS patients, typically due to thrombus formation within the enlarged accessory atrial chamber [4, 18, 24]. Thus, adequate anticoagulation for stroke prevention is necessary, particularly in this patient with newly diagnosed atrial fibrillation and a CHA2DS2-VASc score of 1. The patient was initiated on Edoxaban and adhered well to therapy without reporting any adverse effects or discomfort during follow-up. As he remained asymptomatic and tolerated anticoagulation effectively, no modifications to the treatment plan were required. As reported by the patient, he was surprised by the diagnosis but appreciated the clear explanation and accepted the therapy with trust.

## CONCLUSION

In conclusion, transthoracic echocardiography, cardiac Computed Tomography Angiography (CTA), and virtual endoscopy provide complementary, non-invasive imaging modalities for the diagnosis and anatomical evaluation of CTS. Although adults with CTS are often asymptomatic, they may develop symptoms in the presence of coexisting cardiac conditions. This report presents the first documented case of CTS in combination with chronic adhesive pericarditis, a rare coexistence that may potentially aggravate the hemodynamic effects of CTS and plausibly contribute to the development of atrial fibrillation. Given the increased incidence of atrial fibrillation in adult CTS, anticoagulation is advisable for stroke prevention.

## Data Availability

All data generated or analyzed during this study are included in this published article.

## LIST OF ABBREVIATIONS

CTS: Cor Triatriatum Sinister
CTD: Cor Triatriatum Dextrum
CTA: Computed Tomography Angiography
TTE: Transthoracic Echocardiography
TEE: Transesophageal Echocardiography
EKG: Electrocardiogram
LV: Left Ventricle
ASD: Atrial Septal Defect

## STANDARDS OF REPORTING

CARE guidelines were followed.
